# Augmented Oral Bioavailability and Prokinetic Activity of Levosulpiride Delivered in Nanostructured Lipid Carriers

**DOI:** 10.3390/pharmaceutics14112347

**Published:** 2022-10-31

**Authors:** Sadia Tabassam Arif, Shahiq uz Zaman, Muhammad Ayub Khan, Tanveer A. Tabish, Muhammad Farhan Sohail, Rabia Arshad, Jin-Ki Kim, Alam Zeb

**Affiliations:** 1 Riphah Institute of Pharmaceutical Sciences, Riphah International University, Islamabad 44000, Pakistan; 2 Division of Cardiovascular Medicine, Radcliffe Department of Medicine, University of Oxford, Headington, Oxford OX37BN, UK; 3 Riphah Institute of Pharmaceutical Sciences, Riphah International University Lahore Campus, Lahore 54000, Pakistan; 4 Faculty of Pharmacy, University of Lahore, Lahore 54000, Pakistan; 5 College of Pharmacy, Institute of Pharmaceutical Science and Technology, Hanyang University, 55 Hanyangdaehak-ro, Sangnok-gu, Ansan 15588, Gyeonggi, Korea

**Keywords:** levosulpiride, nanostructured lipid carriers, D-optimal mixture design, oral bioavailability, prokinetic activity, gastric disorders

## Abstract

The present study is aimed to develop and optimize levosulpiride-loaded nanostructured lipid carriers (LSP-NLCs) for improving oral bioavailability and prokinetic activity of LSP. LSP-NLCs were optimized with D-optimal mixture design using solid lipid, liquid lipid and surfactant concentrations as independent variables. The prepared LSP-NLCs were evaluated for physicochemical properties and solid-state characterization. The in vivo oral pharmacokinetics and prokinetic activity of LSP-NLCs were evaluated in rats. LSP-NLCs formulation was optimized at Precirol^®^ ATO 5/Labrasol (80.55/19.45%, *w*/*w*) and Tween 80/Span 80 concentration of 5% (*w*/*w*) as a surfactant mixture. LSP-NLCs showed a spherical shape with a particle size of 152 nm, a polydispersity index of 0.230 and an entrapment efficiency of 88%. The DSC and PXRD analysis revealed conversion of crystalline LSP to amorphous state after loading into the lipid matrix. LSP-NLCs displayed a 3.42- and 4.38-flods increase in AUC and C_max_ after oral administration compared to LSP dispersion. In addition, LSP-NLCs showed enhanced gastric emptying (61.4%), intestinal transit (63.0%), and fecal count (68.8) compared to LSP dispersion (39.7%, 38.0% and 51.0, respectively). Taken together, these results show improved oral bioavailability and prokinetic activity of LSP-NLCs and presents a promising strategy to improve therapeutic activity of LSP for efficient treatment of gastric diseases.

## 1. Introduction

The gastrointestinal (GI) motility problems such as constipation, vomiting and diarrhea have an adverse impact on the quality of life despite that their general consequences are not life-threatening. Dyspepsia or indigestion is associated with heartburn, abdominal discomfort, bloating, nausea, epigastric pain, regurgitation and early satiety [1]. Delayed gastric emptying is one of the major causes of dyspepsia where food stays abnormally longer in the stomach. Chronic diabetes and some prescription medicines also result in reduced gastric emptying [2]. Recently, prokinetic drugs such as dopamine receptor antagonists including metoclopramide, domperidone and levosulpiride (LSP) have been successfully used to treat functional dyspepsia and GI motility disorders [3]. LSP shows its prokinetic activity by antagonizing dopamine D_2_ receptors in the central nervous system as well as in the GI tract and thereby blocks the inhibitory effects of dopamine on GI motility. In addition, LSP has agonistic effects on 5HT_4_ receptors in the enteric nervous system which contributes to its antiemetic effect [4]. LSP has also used to prevent premature ejaculation and as an antipsychotic agent [5].

Despite its merits over other prokinetics, poor bioavailability arising from low aqueous solubility and low permeability (belongs to BCS class IV drug) is a major drawback of oral LSP formulations [5]. The bioavailability of LSP was found to be 23.4% after a single oral administration at a dose of 50 mg in healthy Chinese subjects [6]. Consequently, the amount of LSP reaching the target site from the currently available immediate-release oral tablet is not sufficient to produce optimum therapeutic effects. These problems necessitate the design of strategies to improve its solubilization capacity and oral bioavailability. A number of formulation strategies such as solid dispersions [7], microcapsule [8], solid lipid nanoparticle [5,9] and self-nano-emulsifying drug delivery systems [10] have been investigated for this purpose. However, all the earlier studies are either confined to in vitro dissolution or in vivo pharmacokinetics studies without demonstrating in vivo therapeutic effects of the LPS-loaded carrier system. These studies lack sufficient evidence to indicate the true therapeutic potential of the developed drug delivery system. Among others, lipid-based nanoparticles hold promising applications for efficient oral delivery of poorly water soluble and permeable drugs as they contain biodegradable, biocompatible and safe to use lipids [11]. Moreover, lipid nanoparticles provide sustained drug release, protect the incorporated drugs and biologicals against acidic and enzymatic degradation in GI tract, and enhance drug solubilization and absorption in the intestinal milieu [12]. Lipid nanoparticles are also reported to alter drug transport pathways from the portal to the lymphatic system, thereby reducing their first-pass metabolism and enhancing lymphatic transport [13].

The second generation of lipid nanoparticles “nanostructured lipid carriers (NLCs)” demonstrate merits over their first-generation counterparts “solid lipid nanoparticles (SLNs)” in terms of their physiochemical properties and release behaviors [14]. The imperfections created in the crystal lattice of NLCs by using a binary mixture of solid and liquid lipids provide a high drug payload and prevent drug leakage during long-term storage [15]. Herein, we developed and optimized LSP-loaded NLCs (LSP-NLCs) intending to enhance its solubilization, intestinal absorption and thus oral bioavailability as well as prokinetic activity. LSP-NLCs were prepared with ultrasonication technique and optimized by D-optimal combined mixture design and characterized for physiochemical attributes. Finally, the in vivo pharmacokinetic and prokinetic studies on LSP-NLCs were carried out in rats and compared to those of LSP dispersion.

## 2. Materials and Methods

### 2.1. Materials

Levosulpiride (LSP) was a generous gift from Bio-Labs Pvt. Ltd. (Islamabad, Pakistan). Precirol^®^ ATO 5 and Labrasol^®^ were gifted by Gattefossé (Saint-Priest, France). Tween 80, Span 80, oleic acid, Poloxamer 407, porcine bile extract, pepsin and lipase were purchased from Sigma Aldrich (St. Louis, MO, USA). All other chemicals were of analytical grade and used without further purification.

### 2.2. Selection of Lipids and Surfactants

The solubility of LSP in lipids was evaluated by separately mixing 25 and 50 mg of LSP in 500 mg of solid lipids (Precirol^®^ ATO 5, cetyl alcohol and glyceryl monostearate) or liquid lipids (Labrasol, oleic acid and soybean oil) and heating the mixture at 70 °C. The resultant heated mixture was then observed for clarity and presence of drug particles [16]. Afterwards, solid and liquid lipids showing maximum solubility of LSP were evaluated for their mutual miscibility. For this purpose, they were mixed and heated for 30 min at 75 °C, allowed to cool to room temperature and visually observed for miscibility by checking their clarity, turbidity, uniformity and phase separation.

The selection of suitable emulsifier was carried out by melting a mixture of selected lipids at 75 °C with constant stirring and adding single or a mixture of surfactants and then titrating with distilled water while heating and stirring. The prepared emulsions were observed for clarity and homogeneity [17]. The surfactants examined were Tween 80, Span 80, Poloxamer 407 and propylene glycol.

### 2.3. Experimental Design and Optimization of LSP-NLCs

The effects of compositional factors on the physiochemical properties of LSP-NLCs were determined and optimized by using design expert software (Version 13, Stat-Ease Inc, Minneapolis, MN, USA). A D-optimal combined mixture process design was employed to generate different formulations by using two independent variables consisting of concentration of mixture components (A + B = solid lipid + liquid lipid) and surfactant concentration (C). The particle size, polydispersity index (PDI) and entrapment efficiency of LSP-NLCs were nominated as dependent variables. The concentration range of independent variables and constraints of dependent variables are given in Table 1. The reproducibility of the experimental design was evaluated by replicating formulation combinations.

### 2.4. Preparation of LSP-NLCs

Hot homogenization and ultra-sonication technique was employed for the preparation of LSP-NLCs [18]. Accurately weighed solid and liquid lipids were melted at 70 °C followed by the addition of LSP to the lipid melt to allow its complete dissolution. The aqueous phase was prepared by dissolving surfactants in distilled water and heated up to 70 °C. This hot aqueous phase was then poured into the lipid phase and homogenized at 15,000 rpm for 5 min (Homogenizer HG-15D, DAIHAN Scientific, Wonju, Republic of Korea). The obtained coarse O/W emulsion was further sonicated for 3 min at an amplitude of 50% and a power of 100 W using a probe sonicator at 65 °C (Model VCX750, Sonics and Materials Inc., Newtown, CT, USA). Finally, the obtained nano-emulsion was rapidly cooled down in an ice bath to form LSP-NLCs.

### 2.5. Physicochemical Characterization of LSP-NLCs

Particle size, PDI and zeta potential of LSP-NLCs were measured with a Nanotrac Wave II particle size analyzer (Microtrac MRB, Montgomeryville, PA, USA) based on a dynamic light scattering technique. Samples were suitably diluted with ultra-pure water and the analysis was performed at room temperature.

Spectrophotometric analysis was carried out for the determination of entrapment efficiency and loading content of LSP-NLCs [10]. For this purpose, free LSP and large aggregates were separated by filtration using a syringe filter (0.45 µm) and subsequent centrifugation at 5000 rpm for 10 min. LSP-NLCs deprived of free drug were then dissolved in methanol and the amount of LSP was measured by using UV–visible spectrophotometer (V-530; JASCO Corporation, Tokyo, Japan) at 237 nm. Entrapment efficiency and loading content were determined with the following equations.

$$\mathrm{Entrapment}\;\mathrm{efficiency}\;\left(\%\right)=\frac{\mathrm{amount}\;\mathrm{of}\;\mathrm{LSP}\;\mathrm{loaded}\;\mathrm{in}\;\mathrm{NLCs}\;}{\mathrm{total}\;\mathrm{amount}\;\mathrm{of}\;\mathrm{LSP}\;\mathrm{added}}\times 100$$

$$\mathrm{Loading}\;\mathrm{content}\;\left(\%\right)=\frac{\mathrm{amount}\;\mathrm{of}\;\mathrm{LSP}\;\mathrm{loaded}\;\mathrm{in}\;\mathrm{NLCs}}{\mathrm{total}\;\mathrm{weight}\;\mathrm{of}\;\mathrm{LSP}-\mathrm{NLCs}\;}\times 100$$

Scanning electron microscopy (SEM, VEGA 3 LMU, Tescan Analytics, Brno, Czech Republic) was used to study surface morphology of the optimized LSP-NLCs. After appropriate dilution, a drop of formulation was placed on a sample holder, air dried and imaged at an accelerating voltage of 20 kV [19].

### 2.6. Solid State Characterization of LSP-NLCs

For solid state characterization, the optimized LSP-NLCs formulation was lyophilized without adding any cryoprotectant by using a lyophilizer (TFD5503, IlShin BioBase Co., Ltd., Dongducheon-si, Gyeonggido, Korea). The thermal behavior of lyophilized LSP-NLCs along with other solid components were evaluated by heating accurately weighed samples (3–5 mg) between 30–300 °C in sealed aluminum pans using a differential scanning calorimeter (DSC Q2000; TA Instrument, New Castle, DE, USA). Likewise, the crystalline behavior of these samples was measured at 2θ angel range of 2–80° with a scanning rate of 2°/min by using powder X-ray diffractometer (PXRD, D8, Advance-Bruker, Billerica, MA, USA). The molecular dynamics and interactions among the solid components of LSP-NLCs were evaluated in the wavelength range of 400–4000 cm^−1^ with a Fourier transform infrared spectrophotometer (FTIR, Eco Alpha II-Bruker, Billerica, MA, USA).

### 2.7. In Vitro Drug Release from LSP-NLCs

The in vitro drug release study of LSP-NLCs was carried out by using a dialysis membrane diffusion technique. For this purpose, a dialysis membrane molecular cut-off weight of 10 kDa (Spectrum Laboratories, Inc., Rancho Dominguez, CA, USA) was soaked in the release medium for 20 min. LSP-NLCs and LSP dispersion each equivalent to 5 mg of LSP were taken in the dialysis membrane and immersed in 500 mL of phosphate buffer (pH 6.8) maintained at 37 ± 0.5 °C and continuously stirred at 100 rpm. Tween 80 (0.1%, *w*/*v*) was added to the release media to maintain the sink conditions. Aliquots of the release medium (2 mL) were withdrawn at specified times for 24 h and immediately replaced with fresh phosphate buffer to maintain a constant volume. The obtained samples were filtered using a syringe filter (0.45 µm) and analyzed for the LSP content by using an HPLC system (Shimadzu Nexera LC-40, Shimadzu Scientific Instruments, Kyoto, Japan) equipped with a C_18_ column (4.6 mm × 250 mm × 5 µm, Shim-pack GIST, Shimadzu, Japan), an isocratic pump and a UV detector. HPLC conditions included a mixture of 10 mmol phosphate buffer and methanol (60:40, *v*/*v*) as a mobile phase pumped at a flow rate of 1 mL/min, an injection volume of 30 µL and a column temperature of 30 °C. The eluent was analyzed for an LSP concentration at 237 nm [5]. The chromatogram of HPLC analysis is shown in Supplementary data Figure S1. The in vitro release data was then fitted in different kinetic models such as zero order, first order, Higuchi, Korsmeyer–Peppas and Hixson–Crowell model.

### 2.8. In Vitro Lipid Digestion Study

The in vitro gastro-intestinal behavior and stability of optimized LSP-NLCs was evaluated by using a previously reported simulated digestion method [20]. For this purpose, simulated gastric fluid (SGF) was prepared by dissolving HCl (7 mL, 37% *w*/*w*), NaCl (2 g) and pepsin (3.2 g) in distilled water (1 L) and a pH of SGF was adjusted to 1.2 using 1 M HCl. The simulated intestinal fluid (SIF) was prepared by dissolving pancreatic lipase (4 g/L), bile extract (4.3 g/L) and calcium chloride (0.6 mM) in phosphate buffer saline (PBS), and a pH of SIF was finally adjusted to 7.0 using 1 M NaOH. The digestion study was performed after diluting LSP-NLCs with an equal amount of distilled water.

For gastric phase digestion, 5 mL of diluted LSP-NLCs was mixed with 15 mL of SGF and incubated in a water bath for 2 h at 37 °C with continuous shaking at 100 rpm. Samples were taken and analyzed for particle size and PDI of LSP-NLCs in digestive media. For intestinal phase digestion, 15 mL of LSP-NLCs-incubated gastric phase was mixed with 15 mL of SIF and re-incubated for 2 h at 37 °C with continuous stirring at 100 rpm. The withdrawn samples were again analyzed for their particle size and PDI.

### 2.9. In Vivo Pharmacokinetics

#### 2.9.1. Animals

The in vivo pharmacokinetics studies were conducted in male Sprague Dawley rats (300 ± 20 g) obtained from the animal facility of Riphah International University, Islamabad, Pakistan. The animals were acclimatized with a standardized lab environment for a week before the experiments. All animal studies were in accordance with the NIH policies and animal welfare act and were duly approved by the Research and Ethics Committee of Riphah Institute of Pharmaceutical Sciences (Approval# REC/RIPS/2019/015).

#### 2.9.2. Oral Administration and Blood Collection

Rats were randomly assigned to two groups (*n* = 5) and were kept at fasting for 12 h. A single oral dose of formulations equivalent to 5 mg/kg of LSP was administered through oral gavage. Rats in group one were given LSP dispersion, whereas the other group was administered with LSP-NLCs. Blood samples (0.3 mL) were collected from the jugular vein at the predetermined time intervals of 0.25, 0.5, 1, 2, 3, 4, 6, 8, 12, 16, 20 and 24 h. Plasma was obtained from the blood samples by centrifugation at 3000× *g* for 15 min and was stored at −80 °C until further analysis.

#### 2.9.3. LSP Quantification and Pharmacokinetics Parameters

LSP was extracted from plasma samples by adding 1.5 mL of methylene chloride to the obtained plasma. The resultant mixture was vortexed for 10 min and centrifuged at 3000× *g* for 5 min to separate the organic layer. The organic solvent was then evaporated and the obtained residue was dissolved in 150 μL of mobile phase. Finally, HPLC was used for the quantification of LSP in filtrate according to the method described earlier.

The pharmacokinetic parameters such as area under the plasma concentration–time curve to last time point (AUC_0→t_), area under the plasma concentration–time curve up to time infinity (AUC_0→__∞_), peak plasma concentration (C_max_), time to reach peak plasma concentration (T_max_), half-life (t_1/2_) and elimination constant (K_el_) were determined by a non-compartmental analysis using WinNonlin software (Version 5.2, Scientific Consulting Inc., Apex, NC, USA). The relative bioavailability (F_rel_) of LSP-NLCs was calculated relative to LSP dispersion according to the following equation.

$$F_{\mathrm{rel}}\left(\%\right)=\frac{{\mathrm{AUC}}_{0\to t}\;\mathrm{of}\;\mathrm{LSP}-\mathrm{NLCs}}{{\mathrm{AUC}}_{0\to t}\;\mathrm{of}\;\mathrm{LSP}\;\mathrm{dispersion}}\times 100$$

### 2.10. In Vivo Prokinetic Studies

#### 2.10.1. Gastric Emptying and Intestinal Transit

The in vivo prokinetic effects of LSP-NLCs were evaluated in male Sprague Dawley rats (250 ± 20 g). For this purpose, the gastric emptying rate and the small intestinal transit were measured using phenol red method [21]. The animals were kept on fasting for 24 h before the study, and then randomly divided into control, LSP dispersion and LSP-NLCs groups with 6 rats in each group. Rats in the control group were given normal saline whereas the latter two groups were orally administered with either LSP dispersion or LSP-NLCs at a dose equivalent to 5 mg/kg of LSP. For measuring gastric emptying, the animals subsequently received 1.5 mL of a non-nutrient test meal by oral gavage. The test meal was prepared by dissolving 50 mg of phenol red in 100 mL of carboxy methylcellulose solution (1.5%, *w*/*v*). Three out of six rats in each group were euthanized immediately (t = 0) and the remaining three were sacrificed at 30 min after the administration of a test meal. The stomach and small intestine of each animal were excised by a laparotomy procedure and pylorus and cardiac sphincters were secured by ligation. The stomach was chopped to small pieces, contents were homogenized in 100 mL of 0.1 N NaOH and the resultant homogenate was incubated for 1 h at room temperature. The supernatant (10 mL) was separated and mixed with 1 mL of trichloroacetic acid (33%, *w*/*v*) for the precipitation of proteins. The mixture was then centrifuged at 5000 rpm for 15 min at 25 °C, supernatant was collected and mixed with 2.5 mL of 2N NaOH. Finally, phenol red was quantified by using a spectrophotometric analysis at 560 nm. The gastric emptying rate was determined as the quantity of phenol red in the stomach by using the following equation.

$$\mathrm{Gastric}\;\mathrm{emptying}\;\left(\%\right)=\frac{Y\;-\;X}{Y}\times 100$$

X = Phenol red absorbance at t = 30 min.

Y = Phenol red absorbance at t = 0 min.

For measuring the intestinal transit, the small intestine was separated and its length was measured from pyloric sphincter to the ileocecal junction. The rate of intestinal transit was calculated as the ratio of the distance travelled by the test meal to that of the total length of intestine.

#### 2.10.2. Fecal Excretion

Fecal excretion was measured in separate groups of male Sprague Dawley rats (*n* = 3). The animals were acclimatized for three days in separate grid-floor cages and were fed with normal diet and access to drinking water. Thereafter, food was discontinued and fecal pellets excreted by each rat were collected and counted for a duration of 8, 16 and 24 h. These pellets were checked for wet weight immediately and dry weight after being subjected to drying at 45 °C for 24 h. Any variation in the secretion or reabsorption of intestinal fluids was noted as the ratio of wet to dry fecal weight [21].

### 2.11. Statistical Analysis

The statistical analysis of data was carried out with GraphPad Prism 8 software (version 9.2.0, San Diego, CA, USA) and results are presented as mean ± S.D. To determine the statistical significance among treatment groups, their mean values were compared by employing Student’s *t*-test or a one-way ANOVA with post hoc Dunnett’s test at a significance level of *p* < 0.05.

## 3. Results and Discussion

### 3.1. Preliminary Studies for the Selection of Ingredients

Drug solubility in solid and liquid lipids is one of the major determinants of the loading capacity of NLCs and preventing drug leakage. LSP solubility in different lipids in terms of mixture transparency or turbidity is presented in Supplementary Table S1. Precirol^®^ ATO 5 and Labrasol showed the highest solubilization capacity for LSP among the solid and liquid lipids, respectively, as they allow complete dissolution of LSP by forming transparent solutions. The selected solid and liquid lipids were then subjected to miscibility screening. Precirol^®^ ATO 5 and Labrasol formed a transparent and homogenous solution without any signs of turbidity or phase separation. It is important to mention that miscibility is crucial for the uniform distribution of liquid lipids in the solid lipid matrix to develop stable NLCs [22].

On the other hand, the selection of surfactants was based on their ability to produce clear and stable nano-emulsion. It was observed that combination of Tween 80 and Span 80 produced transparent nano-emulsion, whereas Poloxamer 407 and propylene glycol produced turbid and opaque formulations along with phase separation. It has been reported that the combination of surfactants plays a favorable role in the development of small sized and stable nanoparticles [23].

### 3.2. D-Optimal Combined Mixture Design

A D-optimal combined mixture design was applied to determine the effects of mixture components and surfactant concentrations (independent variables) on the particle size, PDI and entrapment efficiency (dependent variables) of LSP-NLCs. The ratio of solid to liquid lipid was taken as mixture components or Factor 1, whereas the surfactant concentration was taken as Factor 2. Design Expert software generated a total of 22 runs by using a D-optimal combined mixture process design. All these 22 formulations were prepared in triplicate according to the composition devised by software, and the mean values of measured responses are presented in Table 2. The amount of LSP and total lipid in each formulation was 5 and 10 mg, respectively. The ratio of solid lipid was varied between 65–95% and liquid lipid was varied between 5–35% of the total lipid mixture (10 mg or 100%). On the other hand, the surfactant concentration (Tween 80 and Span 80 in equal amount) varied between 2–5% of the total lipid.

The obtained experimental results of each dependent variable were then incorporated in the response column of design expert software and were fitted to an inimitable statistical model to explain the habit of every independent variable. The ANOVA test was applied to find out the adequacy of the proposed experimental model using design expert software, and the results of the statistical analysis are presented in Table 3. The fitted model showed significant model *p*-values of less than 0.05, a non-significant (more than 0.05) lack of fit *p*-values and variability of less than 0.2 among predicted and adjusted R^2^ values for measured responses of all dependent variables. These results show that the arrangement of model space was valid and this model can be employed to navigate design space. 

### 3.3. Effects of Independent Variables on Particle Size, PDI and Entrapment Efficiency

The measured minimum and maximum particle size values among all the design expert-generated formulations were 65.4 nm and 387.2 nm, respectively (Table 2). The relationship between the independent variables and the particle size of LSP-NLCs could be explained on the basis of following polynomial equation generated from the statistical analysis.

(1) Particle Size =+283.76A +66.23B −28.17AC −13.38BC

**Figure 1:**
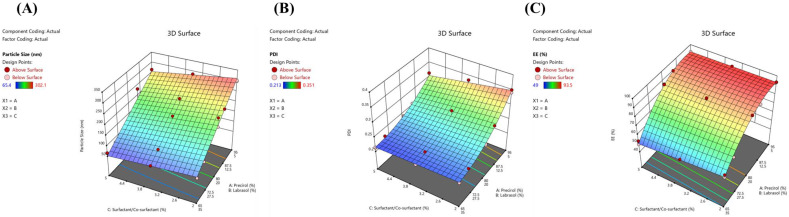
Response surface plots showing the effect of independent variables on particle size (**A**), PDI (**B**) and entrapment efficiency (**C**) of LSP-NLCs within the limits of experimental design.

In the given equation, a positive sign indicates positive effects of the coefficients on response while a negative sign indicates an inverse relationship between the variable and particle size. The influence of a variable on response can be quantified by its magnitude. It is evident that the magnitude of A was much higher than B and C, therefore A is a critical factor in determining the particle size of LSP-NLCs. It signifies that increasing solid lipid amount in NLCs would increase the particle size. Furthermore, the interaction of coefficient A and B with variable C has a negative impact on particle size indicating that an increase in surfactant concentration will decrease particle size. The three-dimensional response surface plot showing the effects of independent variables on the particle size of LSP-NLCs within the limits of the experimental design is presented in Figure 1A. The increase in solid lipid ratio in lipid matrix from 65/35% to 95/5% at same surfactant concentration resulted in an increased particle size of NLCs and vice versa. This drop in NLCs size at higher amounts of liquid lipid could possibly be due to lowered inner phase viscosity of particles and consequently reduced surface tension to generate smaller and smoother particles [24]. On the other hand, particle size was also reduced as the surfactant concentration was increased from 2% to 5%, which might be due to reduced interfacial tension between aqueous and lipid phases thus forming smaller emulsion droplets and NLCs [25]. Higher surfactant concentration might also prevent the coalescence of small particles into larger ones by providing steric hindrance [26].

All prepared formulations of LSP-NLCs showed homogeneous and narrow particle size distribution as their measured PDI values were in the range of 0.213–0.351. The relationship among independent variables and their effects on PDI is schematically illustrated by a three-dimensional response surface plot (Figure 1B). The statistical analysis generated a polynomial equation for PDI showing A, B, AB, and AC as significant model terms (*p* < 0.05).

(2) PDI =+0.3274A +0.2182B −0.1001AB −0.0220AC −0.0029BC +0.156ABC

The response surface plot and the equation show that increasing the solid lipid content in the lipid matrix directly effects the PDI of LSP-NLCs as a higher PDI is obtained at higher solid lipid concentration. The addition of liquid lipid to the matrix prevents crystallization of solid lipid thereby decreasing PDI values [27]. On the other hand, PDI decreases with increase in surfactant concertation (2% vs. 5%) which might be due to the formation of stable, small size and uniform emulsion droplets [28]. It has been reported that at higher surfactant concentration, a sufficient number of surfactant molecules cover the hydrophobic particles, thereby decreasing interfacial tension, preventing coalescence of nano-emulsion droplets and forming smaller NLCs [29].

Lastly, the effects of independent variables on entrapment efficiency and their inter-relationship are shown graphically in Figure 1C and mathematically by the following equation.

(3) Entrapment efficiency=+92.91A+53.55B+51.50AB−0.4544AC−3.81BC+1.06ABC

Terms A, B, AB, BC were established as significant model terms for entrapment efficiency with *p*-values less than 0.05. The entrapment efficiency of LSP-NLCs increased from 49% to 93.5% as the amount of Precirol^®^ ATO 5 was increased (65% vs. 95%). This increase in entrapment efficiency could be attributed to lipophilic nature of LSP that favors affinity towards lipid matrix of NLCs. Furthermore, it was also observed that an increase in surfactant concentration to 5% resulted in a decrease in the entrapment efficiency. It might be explained on the basis of partition phenomenon where a higher surfactant concentration increases solubilization of drug in external aqueous medium. It results in reduced drug portioning to the external phase and thereby lowers the entrapment efficiency of LSP-NLCs [30].

### 3.4. Optimization of LSP-NLCs

**Figure 2:**
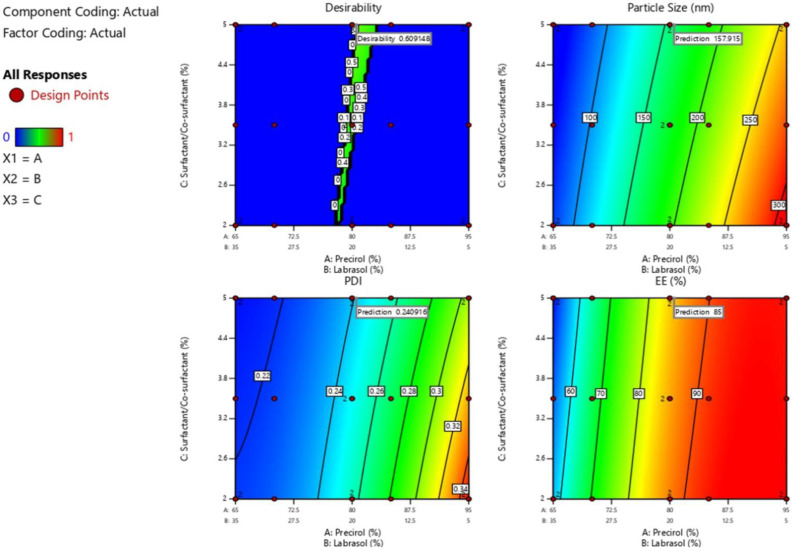
Contour plots of desirability and predicted responses for optimized LSP-NLCs formulation.

The desirability function in design expert software was used for the numerical optimization to achieve final LSP-NLCs formulation. The selected goal for independent variables was set to in-range with importance level of three. On the other hand, the designated goals were set to minimize for particle size and in-range for PDI and entrapment efficiency at importance level of five. The selected ranges for PDI and entrapment efficiency were 0.2–0.25 and 85–95%, respectively. A total of six compositions were generated by the design expert software and composition with maximum desirability of 0.609 was selected as optimized formulation. The software-generated composition for optimized LSP-NLCs was 80.55% Precirol^®^ ATO 5, 19.45% Labrasol and 5% Tween 80/Span 80 mixture. The predicted particle size, PDI and entrapment efficiency of optimized LSP-NLCs were 157.9 nm, 0.241 and 85%, respectively. The contour plots of desirability and predicted responses for optimal formulation are shown in Figure 2. The optimized formulation was prepared in triplicate according to composition suggested by software and obtained values of particle size, PDI and entrapment efficiency are presented in Table 3. The accuracy of the model was examined by measuring % difference between experimentally obtained and model-predicted values for each response. A very low error (<5%) was observed for each response showing the closeness of obtained and predicted values. Taken together, these results indicate the validity of the employed model and therefore the successful optimization of LSP-NLCs.

### 3.5. Physicochemical Properties of Optimized LSP-NLCs

**Figure 3:**
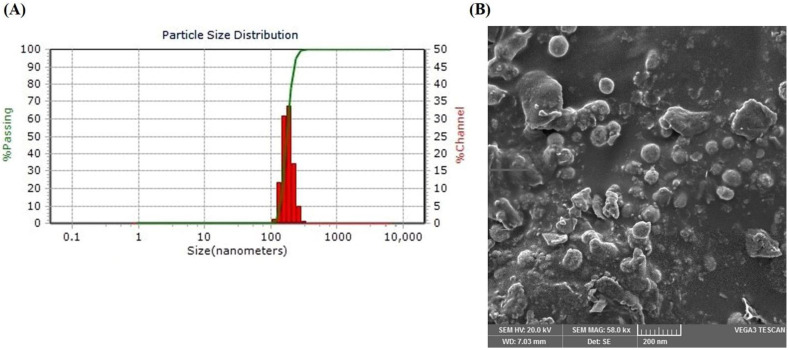
Particle size distribution curve (**A**) and surface morphology indicated by SEM micrographs (**B**) of the optimized LSP-NLCs.

The particle size, PDI, zeta potential and drug loading of nanoparticles are the major determinants of their overall therapeutic performance. The optimized LSP-NLCs demonstrated particle size and PDI of 152.0 ± 4.7 nm and 0.230 ± 0.05, respectively. It is generally agreed that nanoparticles with a particle size of less than 300 nm show efficient intestinal absorption into the lymphatic system and thus improve bioavailability after oral administration [31]. Furthermore, the low PDI value coupled with a unimodal and narrow size distribution curve of optimized LSP-NLCs (Figure 3A) indicate their homogeneity which is crucial for their uniform in vitro and in vivo behavior. The generally acceptable limit of PDI to indicate homogeneous dispersion of nanoparticles is less than 0.3 [32]. The optimized LSP-NLCs were negatively charged with adequate zeta potential of −17.9 ± 1.0 mV to ensure colloidal stability of the formulation. Previously, a zeta potential of around −20 mV has been proven enough to stabilize lipid-based nanoparticles upon storage [33]. Entrapment efficiency is another important property that reduces the volume of formulation to be administered during animal studies. The optimized LSP-NLCs showed a very high entrapment efficiency of 88.0 ± 2.4% with a drug loading content of 6.4 ± 1.3%, which could be ascribed to a less ordered imperfect structure of NLCs due to the incorporation of liquid lipid in their matrix [34]. Morphological features of LSP-NLCs revealed by the SEM image (Figure 3B) demonstrated distinct and homogeneously distributed nanoparticles with spherical shape with smooth surfaces. Based on these satisfactory physicochemical properties, the optimized LSP-NLCs were subjected to further in vitro evaluation.

### 3.6. DSC, PXRD and FTIR Analysis

**Figure 4:**
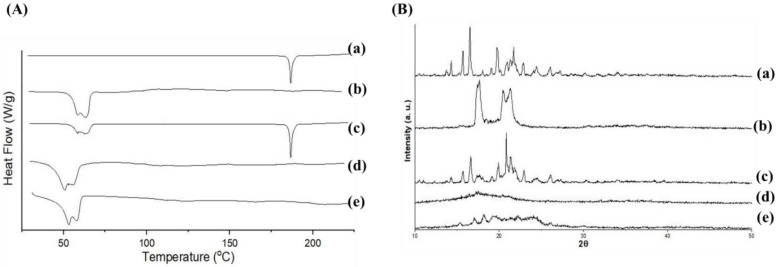
DSC thermograms (**A**) and PXRD patterns (**B**) of LSP (a), Precirol ATO 5 (b), physical mixture of LSP and Precirol ATO 5 (c), blank NLCs (d) and LSP-NLCs (e).

Thermal behavior of lyophilized LSP-NLCs and their individual components were monitored to determine their polymorphic state upon incorporation in the lipid matrix and results are presented as DSC thermograms in Figure 4A. A sharp endothermic peak was observed at 186.6 °C in a DSC thermogram of pure LSP (a) which corresponds to its melting point and indicates the crystalline nature of LSP. Similarly, Precirol^®^ ATO 5 showed its crystalline peak in the range of 59–64 °C (b), whereas the physical mixture of LSP and Precirol^®^ ATO 5 presented their respective individual peaks in the DSC pattern (c). The crystalline endothermic peak of LSP was vanished in thermograms of blank NLCs (d) and LSP-NLCs (e) and only peaks for Precirol^®^ ATO 5 were observed at a slightly reduced melting temperature compared to bulk lipid (b). This small drop in melting temperature of lipid matrix can normally be attributed to the nanosize of prepared NLCs (kelvin effect) with some contribution from lipid interaction with surfactants [35]. The disappearance of LSP peak in LSP-NLCs showed its complete solubilization in lipid matrix with subsequent conversion to an amorphous state [36].

The polymorphic transition of drug within the lipid matrix was further confirmed with PXRD patterns (Figure 4B). Bulk LSP (a) displayed a crystalline nature characterized by its sharp and intense peaks at 2θ scattered angles of 12.8°, 14.5°, 16.5°, 17.1°, 18.1°, 20°, 21.2°, 22.2°, 23.6°, 24.1°, 24.7°, 26.1°, 28.1°, 30.2°, 31.6°, 35.2° and 40.2°. Likewise, the intrinsic sharp peaks for Precirol^®^ ATO 5 (b) were observed at 19.6°, 22.4°, 23.1°, 24.2° and 35.6°. All the major characteristic peaks of LSP and Precirol^®^ ATO 5 were present in the diffractogram of their physical mixture (c). On the other hand, the intrinsic peaks of LSP vanished in the diffraction pattern of LSP-NLCs (e) and its shape was quite similar to that of blank NLCs (d). The results of the PXRD analysis also confirmed that the crystalline LSP was converted into amorphous form when loaded into NLCs and overall reduction in the crystallinity of Precirol^®^ ATO 5 in the lipid matrix. These results also suggest the complete and successful incorporation of drug in the lipid core of NLCs.

**Figure 5:**
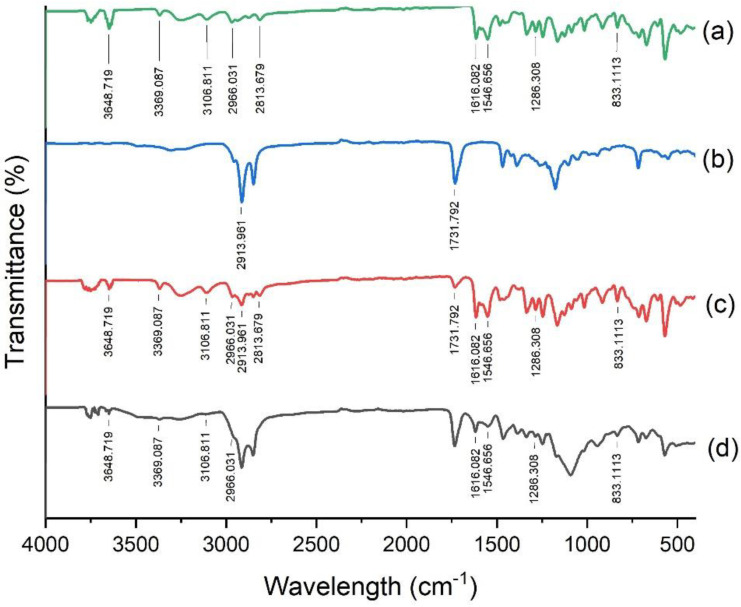
FTIR spectra of LSP (**a**), Precirol ATO 5 (**b**), physical mixture of LSP and Precirol ATO 5 (**c**) and LSP-NLCs (**d**).

FTIR analysis was done to investigate the molecular interactions between LSP and formulation ingredients and their chemical characteristics. The IR spectra of pure LSP, Precirol^®^ ATO 5, their physical mixture and LSP-NLCs were obtained in 4000-400 cm^−1^ and shown in Figure 5. The vibration of LSP at 3648.71 cm^−1^ corresponds to N−H of amide, 3369.08 cm^−1^ for sulfonamide bond, and 3106.81 cm^−1^ for aromatic functional group (a). The peaks for C−H of methylene and methyl functional group were found at 2966.03 cm^−1^ and 2813.67 cm^−1^, C=O stretching of amide group at 1616.82 cm^−1^, C=C (aromatic) bending at 1546.65 cm^−1^, respectively. The vibration at 1286.3 cm^−1^ represented C-O (methoxy) and 833.11cm-1 was observed for C-H (aromatic) groups, correspondingly. Precirol^®^ ATO 5 presented peaks for C=O stretching at 1731.79 cm^−1^ and C-H stretching at 2913.96 cm^−1^ (b). All the characteristic peaks for LSP were found in the spectrum of LSP-NLCs and the physical mixture, which demonstrated compatibility of LSP with the formulation ingredients [10].

### 3.7. In Vitro Drug Release

**Figure 6:**
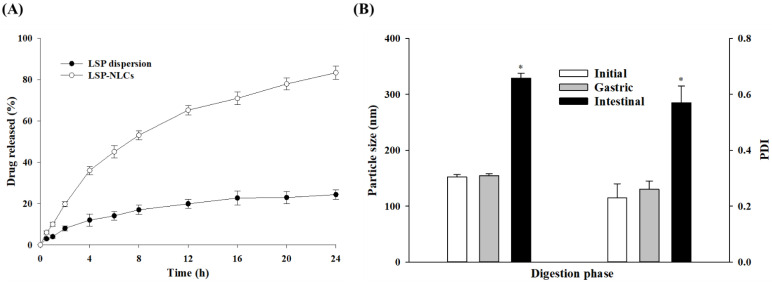
In vitro drug release from LSP-NLCs and LSP dispersion (**A**) and effects of incubation in gastric and intestinal phases on mean particle size and PDI of optimized LSP-NLCs (**B**) Data are presented as mean ± S.D. (*n* = 3). * *p* ˂ 0.001 against their respective initial values.

Comparative evaluation of in vitro drug release from the optimized LSP-NLCs and LSP dispersion was performed. The cumulative release from each formulation was plotted against the respective time interval as shown in Figure 6A. LSP-NLCs exhibited a sustained drug release pattern for 24 h with a slightly higher release rate in the initial 4 h followed by a slower release. LSP-NLCs showed a cumulative release of 36.0 ± 2.0% and 83.3 ± 3.1% in 4 and 24 h, respectively. On the other hand, LSP dispersion displayed only a 24.3 ± 2.3% release in 24 h. The initial faster release from LSP-NLCs could be ascribed to the release of drug accumulated in the outer, softer liquid-lipid rich part of the lipid matrix. This liquid-lipid rich part has relatively higher drug solubility for lipophilic drugs and generates a high gradient for an initial faster release [15]. The subsequent release from the central part of LSP-NLCs is much more uniform and is mostly driven by diffusion and matrix degradation. Contrastingly, the low aqueous solubility of crystalline LSP might be the main reason for hindered drug release from dispersion reaching out to be only 24.3% after 24 h [37]. LSP exists in crystalline form in dispersion as confirmed by DSC and PXRD, therefore the high lattice energy of stable crystals reduces its solubilization and subsequent release from dispersion.

The release kinetics process from LSP-NLCs was evaluated by fitting the data from in vitro release into various kinetic models such as zero order, first order, Higuchi, Korsmeyer–Peppas and Hixson–Crowell model. The obtained results of correlation coefficients (R^2^) and release exponents (n) are presented in Table 4. The results show that the release from LSP- NLCs was best fitted with Korsmeyer–Peppas kinetic model. The release exponent (n) value greater than 0.45 in Korsmeyer–Peppas kinetic model indicated the non-Fickian anomalous diffusion [38]. These results suggest that drug release from LSP-NLCs is mainly governed by LSP diffusion and erosion of lipid matrix [39]. The mechanism of diffusion and erosion processes is also affected by the lipid matrix composition and type of surfactant. High molecular weight PEG moiety of Labrasol could slow diffusion of LSP via a barrier formation on NLCs surface [29]. On the other hand, Tween 80 produces steric stabilization effect could be expected to slow lipid matrix degradation [40].

### 3.8. Lipid Digestion in Gastric and Intestinal Fluids

The lipid digestion study was performed to assess the stability of LSP-NLCs in different segments of the GI tract because the acidic gastric environment could destabilize NLCs, adversely affect the drug release and absorption after oral administration. For these reasons, particle size and PDI values before and after incubation with simulated gastric and intestinal fluids were monitored and compared (Figure 6B). The results indicated that LSP-NLCs were firmly stable after 2 h of incubation in simulated gastric fluid as they retained their particle size (154. 7 ± 3.2 nm) and PDI (0.26 ± 0.03) compared to the initial values (152.0 ± 4.7 nm, 0.23 ± 0.05). This stability of LSP-NLCs in hydrolyzing acidic environment could possibly be attributed to the steric stabilization provided by nonionic surfactants (Tween 80 and Span 80), thereby preventing their hydrolysis by proteases and making them resistant to aggregation and degradation [41,42]. The stability of Precirol^®^ ATO 5 and Tween 80-based lipid nanoparticles in gastric environment has also been previously reported [18,43]. In contrary to the stability in gastric fluid, LSP-NLCs showed 2.16- and 2.48-fold increases in their particle size and PDI compared to the initial values (328.7 vs. 152.0 nm, 0.57 vs. 0.23), respectively. LSP-NLCs aggregation (increase particle size) in intestinal fluid might be due the removal of the surfactant layer by pancreatic lipase or bile salts, thus predisposing their surface to further hydrolysis by digestive enzymes [44,45]. Pancreatic lipases digest lipid-based drug delivery systems to form secondary structures or mixed micelles which in turn solubilize drugs with poor aqueous solubility [46,47]. Some of the major constituents of mixed micelles are phospholipids, bile salts and products of lipid digestion such as fatty acids and monoglycerides. This process is expected to enhance LSP solubility and bio-accessibility at the absorption site [48]. Collectively, these findings suggest the stability of LSP-NLCs in stomach and their suitability for absorption in the intestine.

### 3.9. Pharmacokinetics of LSP-NLCs

**Figure 7:**
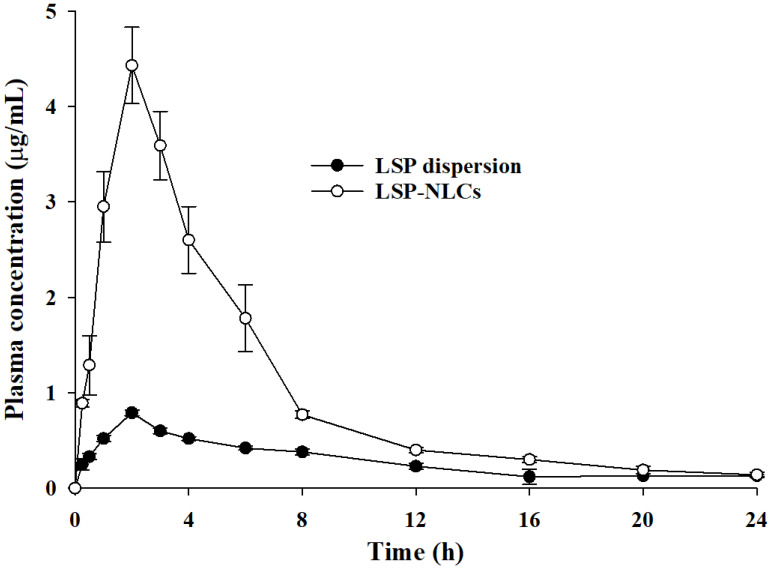
Average plasma drug concentration vs. time curves obtained after oral administration of LSP-NLCs and LSP dispersion to rats at a dose equivalent to 5 mg/kg of LSP. Data are expressed as mean ± S.D. (*n* = 3 for LSP dispersion, *n* = 5 for LSP-NLCs).

LSP possesses poor oral bioavailability owing to its low water solubility, dissolution and absorption. The oral pharmacokinetics profile of the developed LSP-NLCs was evaluated and compared to those of LSP dispersion in rats. The resultant mean plasma concentration–time curves of each formulation are presented in Figure 7 and the calculated pharmacokinetics parameters are tabulated in Table 5. The results indicated that LSP-NLCs exhibited a substantially higher plasma concentration at each time interval compared to LSP dispersion. Furthermore, it was evidenced that the key parameters to describe oral bioavailability including AUC_0→t_, AUC_0→__∞_ and C_max_ were 3.42-, 3.04- and 4.38-times higher for LSP-NLCs as compared to LSP dispersion, respectively. The relative bioavailability of LSP-NLCs was 339.18% compared to LSP dispersion. These results show better absorption and bioavailability of LP-NLCs than LSP dispersion in the GI tract after oral administration. However, other pharmacokinetics parameters such as T_max_, t_1/2_ and K_el_ were not significantly different between LSP-NLCs and LSP dispersion. The improved absorption and bioavailability of LSP-NLCs can be attributed to the effectively increased surface area facts of small size NLCs and improved LSP solubility due to its conversion to amorphous state [12,49]. Increased surface area has also been reported to influence the adhesion of NLCs to the GI tract, thereby increasing their contact time for better absorption [50]. Additionally, the presence of the fatty acid chain in Precirol^®^ ATO 5 improves lymphatic transport and uptake of lipid nanoparticles and reduce first pass effect of the drugs [51,52]. Collective contributions from all these factors resulted in an enhanced oral bioavailability of LSP-NLCs.

### 3.10. Prokinetic Activity of LSP-NLCs

**Figure 8:**
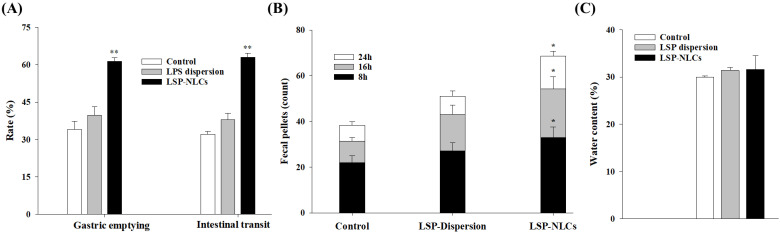
Effect of optimized LSP-NLCs on the gastric emptying and intestinal transit rates (**A**), fecal output (**B**) and water contents (**C**) in rats. Data are presented as mean ± S.D. (*n* = 3). * *p* ˂ 0.05 and ** p ˂ 0.01 against control group.

The therapeutic effects arising from the enhanced oral bioavailability of LSP-NLCs were further conformed by evaluating in vivo prokinetic activity. For this purpose, gastric emptying and intestinal transit rates for a non-nutrient meal of phenol red dye-coated carboxymethyl cellulose were determined in rats. Rodents such as rats and mice are widely-utilized small experimental animals for physiological assessments in GI tract disorders. The use of phenol red test dye in living rats provides better assessment of their GI motility than anesthetized rats with suppressed motor activity due to anesthesia [53]. The obtained results for gastric emptying and intestinal transit rates are presented in Figure 8A. The results showed that LSP-NLCs treatment significantly increased the gastric emptying rate to 61.4 ± 1.4% compared to LSP dispersion (39.7 ± 3.4%) and control (34.1 ± 3.2%) groups. Similarly, the intestinal transit rate calculated as % ratio of distance travelled by the test meal to the total length of intestine was also significantly increased for LSP-NLCs (63.0 ± 1.7%) compared to LSP dispersion (38.0 ± 2.6%) and control (32.1 ± 1.1%) rats. Gastric emptying and intestinal transit are important physiological functions of GI tract and prokinetic drugs are commonly used to treat motility disorders. For these reasons, gastric emptying and intestinal transit are well-established indicators to evaluate relative effectiveness of prokinetic drugs and formulations in animal models [54,55]. In this context, increased gastric emptying and intestinal transit after treatment with LSP-NLCs indicated their better prokinetic activity compared to unformulated LSP.

The prokinetic effects of LSP-NLCs were further assessed by determining the fecal output of rats after 8, 16 and 24 h (Figure 8B). The results indicated that fecal count of rats was significantly higher for LSP-NLCs at each time interval when compared to control and LSP dispersion-treated rats. After 24 h, fecal pellets count and weight of wet stool for LSP-NLCs group were 68.6 ± 2.1 and 12.1 ± 1.0 g (*n* = 3), respectively. In contrary, fecal output and weight of wet stool were substantially lower for LSP dispersion (51.0 ± 2.4, 9.2 ± 1.8 g) and the control group (38.3 ± 1.6 and 7.4 ± 1.3 g). Increased fecal pellets count and weight in LSP-NLCs-treated rats could also be attributed to improved GI motility, peristalsis and fecal excretion, and validates their better prokinetic activity compared to LSP dispersion and control groups [56]. Lastly, water contents (%) were calculated from the fecal excretion data and the results are shown in Figure 8C. There was no significant change observed in water content between LSP-NLCs (31.6 ± 2.9%), LSP dispersion (31.4 ± 0.6%) and control groups 30.0 ± 0.2%). The absence of any effect on water content of feces indicates that LSP-NLCs do not have a significant secretory effect on GI tract. Collectively, these outcomes revealed improved prokinetic effects of LSP-NLCs characterized by increased gastric emptying, intestinal transit and fecal excretion. This enhanced prokinetic activity could be attributed to improved absorption of LSP from LSP-NLCs, thereby successfully blocking the enteric (neuronal and muscular) dopamine D_2_ receptors along with mild antagonist 5-HT_3_ and weak partial agonist effect at the 5-HT_4_ receptors [57]. In addition, the results of in vivo pharmacokinetics are well reflected and correlated with those of in vivo prokinetic effects. 

## 4. Conclusions

Poor oral bioavailability of LSP is one of the major challenges in its successful utilization as an effective prokinetic drug. We herein successfully optimized LSP-NLCs with a D-optimal mixture design and formulated them with hot homogenization and an ultra-sonication technique. The optimized LSP-NLCs showed suitable physicochemicals and exhibited stability in simulated gastric fluid. The in vivo oral bioavailability of LSP was also significantly improved when delivered as LSP-NLCs. Additionally, the prokinetic study revealed that the optimized LSP-NLCs formulation significantly enhanced the gastric emptying, intestinal transit and fecal output in rats. These findings suggest that NLCs could be promising carriers to improve the suboptimal therapeutic effects of poorly water-soluble drugs used to treat gastric disorders.

## Figures and Tables

**Table 1:** Concentration levels of independent variables and response constraints of dependent variables.

Independent Variables		Lower Level	Upper Level
**Factor 1 (A+B)**	A = solid lipid	65%	95%
	B = liquid lipid	5%	35%
**Factor 2**	C = surfactant	2%	5%
**Dependent variables**		Constraints	
**Response 1**	Particle size (nm)	Minimum	
**Response 2**	PDI	In range (0.2–0.25)	
**Response 3**	Entrapment efficiency (%)	In range (85–95%)	

**Table 2:** D-optimal design generated experimental runs for the optimization of LSP-NLCs and measured values of independent variables.

	Factor 1 (A + B)		Factor 2	Response 1	Response 2	Response 3
Run	A: Precirol (%)	B: Labrasol (%)	C: Tween 80/Span 80 (%)	Particle Size (nm)	PDI	Entrapment Efficiency (%)
**1**	65	35	2	74.2 ± 5.3	0.219 ± 0.011	57.0 ± 6.4
**2**	65	35	2	75.0 ± 3.7	0.220 ± 0.027	60.0 ± 4.9
**3**	65	35	5	65.4 ± 7.7	0.213 ± 0.020	49.0 ± 7.7
**4**	65	35	5	66.6 ± 8.3	0.215 ± 0.023	53.0 ± 5.6
**5**	65	35	3.5	67.4 ± 6.2	0.216 ± 0.032	54.0 ± 4.1
**6**	70	30	2	106.3 ± 4.6	0.234 ± 0.021	67.2 ± 7.5
**7**	70	30	5	78.1 ± 5.2	0.224 ± 0.024	61.0 ± 3.0
**8**	70	30	3.5	103.2 ± 4.1	0.228 ± 0.027	63.5 ± 5.2
**9**	80	20	5	106.7 ± 5.3	0.235 ± 0.031	84.5 ± 6.9
**10**	80	20	2	188.7 ± 6.8	0.249 ± 0.019	89.3 ± 5.2
**11**	80	20	3.5	165.3 ± 8.9	0.240 ± 0.029	87.9 ± 6.4
**12**	80	20	3.5	186.1 ± 9.3	0.244 ± 0.028	88.6 ± 3.7
**13**	80	20	5	123.4 ± 4.5	0.239 ± 0.011	85.0 ± 7.2
**14**	80	20	2	231.6 ± 8.1	0.253 ± 0.029	89.6 ± 8.8
**15**	85	15	3.5	234.6 ± 6.3	0.283 ± 0.014	90.2 ± 2.9
**16**	85	15	2	236.1 ± 9.4	0.286 ± 0.022	90.5 ± 5.9
**17**	85	15	5	232.5 ± 7.2	0.256 ± 0.016	90.0 ± 3.8
**18**	95	5	2	298.4 ± 6.1	0.342 ± 0.023	93.2 ± 2.6
**19**	95	5	5	247.0 ± 5.4	0.292 ± 0.019	92.1 ± 3.1
**20**	95	5	2	302.1 ± 9.3	0.351 ± 0.020	93.5 ± 2.9
**21**	95	5	3.5	387.2 ± 7.6	0.336 ± 0.016	93.0 ± 2.5
**22**	95	5	5	265.2 ± 8.3	0.314 ± 0.023	92.3 ± 3.4

The amount of LSP was 5 mg in all formulations. The concentration of Precirol^®^ ATO 5, Labrasol, Tween 80 and Span 80 are shown in % *w*/*w*. Responses are presented as mean ± S.D. (*n* = 3).

**Table 3:** Summarized results of statistical analysis of experimental data with ANOVA showing values of fitted models for dependent variables along with predicted and obtained outcomes of optimized LSP-NLCs.

Response	F-Value	Model *p*-Value	Lack of Fit	Lack of Fit *p*-Value	R^2^	Adjusted R^2^	Predicted Mean Value	Obtained Value	Error (%)
**Particle size (nm)**	103.83	<0.0001	3.03	0.0756	0.9454	0.9363	157.9	152.0 ± 4.7	3.7
**PDI**	139.21	<0.0001	1.50	0.3039	0.9775	0.9705	0.240	0.230 ± 0.05	4.2
**Entrapment efficiency (%)**	240.21	<0.0001	3.58	0.0533	0.9869	0.9827	85.0	88.0 ± 2.4	3.5

The obtained values are presented as mean ± S.D. (*n* = 3). Error (%) = (obtained value–predicted value)/predicted value × 100.

**Table 4:** The correlation coefficient (R^2^) and release exponent (*n*) values obtained by fitting in vitro release data for LSP-NLCs in various release kinetic models.

Kinetic Model	R^2^	*n*
Zero order	0.7686	-
First order	0.9809	-
Higuchi	0.9816	-
Korsmeyer–Peppas	0.9834	0.533
Hixson–Crowell	0.9464	-

**Table 5:** Pharmacokinetic parameters in rat plasma following oral administration of LSP-NLCs and LSP dispersion at a dose equivalent to 5 mg/kg of LSP.

Parameters	LSP Dispersion	LSP-NLCs
AUC_0→t_ (μg∙h/mL)	7.14 ± 0.09	24.44 ± 2.52 **
AUC_0→∞_ (μg∙h/mL)	8.55 ± 0.07	25.98 ± 3.03 **
C_max_ (µg/mL)	1.01 ± 0.02	4.43 ± 0.50 **
T_max_ (h)	1.5 ± 0.00	2.00 ± 0.00
t_1/2_ (h)	7.97 ± 1.16	7.42 ± 1.09
K_el_	0.09 ± 0.01	0.09 ± 0.01
F_rel_ (%)	---	339.18 ± 26.17

AUC_0→t_: area under the plasma concentration-time curve from time zero to time t, AUC_0→__∞_: area under the plasma concentration–time curve from time zero to an infinite time, C_max_: maximum plasma concentration, T_max_: time to reach C_max_, t_1/2_: half-life, K_el_: elimination rate constant, F_rel_: relative bioavailability. Data are presented as mean ± S.D. (*n* = 3 for LSP dispersion, *n* = 5 for LSP-NLCs). ** p *˂* 0.01 against LSP dispersion.

## Supplementary Materials

The following supporting information can be downloaded at: https://www.mdpi.com/article/10.3390/pharmaceutics14112347/s1, Figure S1: HPLC chromatograms of different concentrations of LSP in mobile phase; Table S1: Solubility of LSP in melted solid lipids and liquid lipids heated at 75 °C evaluated in terms of solution transparency (soluble) or turbidity (insoluble).
