# Supplementary material for: Augmented Oral Bioavailability and Prokinetic Activity of Levosulpiride Delivered in Nanostructured Lipid Carriers
Source: Pharmaceutics. 2022 Oct 31;14(11):2347. doi: 10.3390/pharmaceutics14112347 (PMC9695558; doi:10.3390/pharmaceutics14112347)
Supplement: Supplementary file 1 [file pharmaceutics-14-02347-s001.zip › pharmaceutics-1989233-supplementary.pdf]

Supplementary Table S1. Solubility of LSP in melted solid lipids and liquid lipids heated at 75ºC evaluated in terms of solution transparency (soluble) or turbidity (insoluble).

	**Lipid**	**LSP amount**	
		**25 mg**	**50 mg**
**Solid lipids**	Precirol® ATO5	transparent	transparent
	Cetyl alcohol	transparent	turbid
	Glyceryl monostearate	turbid	turbid
**Liquid lipids**	Labrasol®	transparent	transparent
	Oleic acid	turbid	turbid
	Soybean oil	transparent	turbid

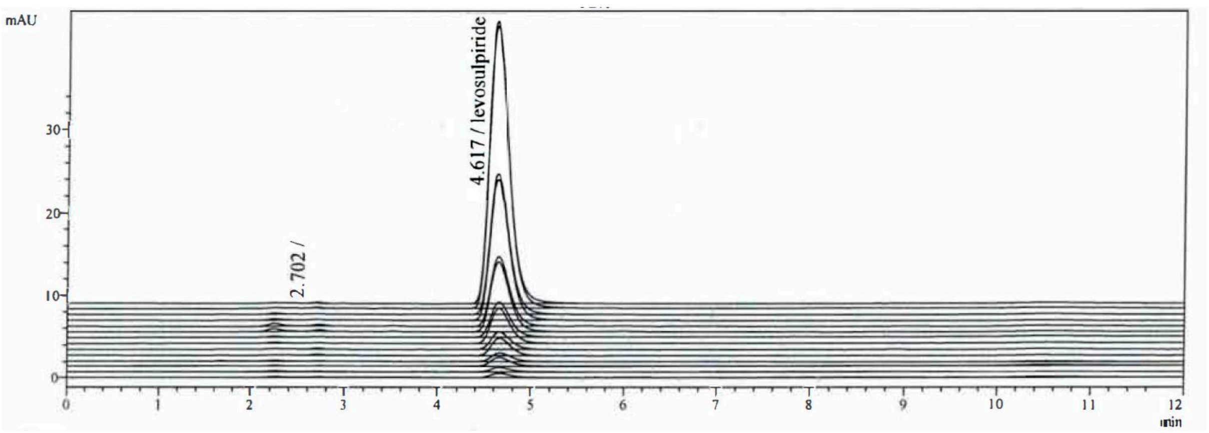

Supplementary Figure S1. HPLC chromatograms of different concentrations of LSP in mobile phase.
